# Consensus Guidance for Genetic Counseling in *GBA1* Variants: A Focus on Parkinson's Disease

**DOI:** 10.1002/mds.30006

**Published:** 2024-09-11

**Authors:** Sophia R.L. Vieira, Roxana Mezabrovschi, Marco Toffoli, Sara Lucas Del Pozo, Elisa Menozzi, Stephen Mullin, Selen Yalkic, Naomi Limbachiya, Sofia Koletsi, Nadine Loefflad, Grisel J. Lopez, Ziv Gan‐Or, Roy N. Alcalay, Ellen Sidransky, Anthony H.V. Schapira

**Affiliations:** ^1^ Department of Clinical and Movement Neurosciences University College London Queen Square Institute of Neurology London United Kingdom; ^2^ Faculty of Health University of Plymouth Plymouth United Kingdom; ^3^ Aligning Science Across Parkinson's Collaborative Research Network Chevy Chase Maryland USA; ^4^ Medical Genetics Branch, National Human Genome Research Institute, National Institutes of Health Bethesda Maryland USA; ^5^ Department of Neurology and Neurosurgery, The Neuro (Montreal Neurological Institute‐Hospital), and Department of Human Genetics McGill University Montreal Quebec Canada; ^6^ Columbia University Irving Medical Center New York New York USA; ^7^ Tel Aviv Sourasky Medical Center, Tel Aviv School of Medicine, Tel Aviv University Tel Aviv Israel

**Keywords:** GBA1, Parkinson's, counseling, neurodegeneration, risk

## Abstract

Glucocerebrosidase (*GBA1*) variants constitute numerically the most common known genetic risk factor for Parkinson's disease (PD) and are distributed worldwide. Access to *GBA1* genotyping varies across the world and even regionally within countries. Guidelines for *GBA1* variant counseling are evolving. We review the current knowledge of the link between *GBA1* and PD, and discuss the practicalities of *GBA1* testing. Lastly, we provide a consensus for an approach to counseling people with *GBA1* variants, notably the communication of PD risk. © 2024 The Author(s). *Movement Disorders* published by Wiley Periodicals LLC on behalf of International Parkinson and Movement Disorder Society.

## The Value of Genomic Information

There is a growing utilization of genetic testing in modern medicine.^1^ New technologies are better equipped to meet the demand for large‐scale identification of genetically homogenous patient subgroups for evaluation in clinical trials and implementation of targeted therapies.^1^ Addressing the meaning of the emerging genomic information available for patients and their families is more important than ever and increasingly complex.

One surprising finding to emerge from the exponential increase in genetic studies was that carrier status of certain early‐onset autosomal recessive diseases, previously considered a benign state, may in fact be associated with adult‐onset related or unrelated disorders.^2^ Heterozygotes of glucocerebrosidase (*GBA1*) variants, the gene implicated in the lysosomal storage disorder (LSD) Gaucher's disease (GD) in biallelic form, constitute the most important genetic risk factor for Parkinson's disease (PD).^2^ PD, the second most common neurodegenerative disorder worldwide, is defined by bradykinesia, rigidity (parkinsonism), and associated tremor.^2^ GD, the second most common LSD after Fabry's disease, is defined by the accumulation of glucosylceramide‐loaded macrophages in visceral organs and bone marrow stromal cells, manifesting with variable symptomatology, typically hepatosplenomegaly, anemia, thrombocytopenia, and bone involvement.^2^ PD phenotype and disease course are influenced by the presence and severity of both GD pathogenic and non‐GD pathogenic *GBA1* variants, which exhibit an average prevalence rate of 5% to 20% within the PD population.^2^, ^3^ *GBA1* variants have also been associated with a higher risk of dementia with Lewy bodies; however, the focus of this review will be on its link with PD.^4^

Genetic testing and counseling for PD patients with *GBA1* variants and nonmanifesting *GBA1* carriers (typically first‐degree relatives of GD patients or index *GBA1* variant carriers) remain largely limited to specialist neurology centers and research settings. Notably, access to *GBA1* genotyping varies across the world.^5^ Recent initiatives, ROPAD (active recruiting; ClinicalTrials.gov Identifier: NCT03866603), PD GENEration (ClinicalTrials.gov Identifier: NCT04057794), and others have improved access to *GBA1* testing in many countries.^5^, ^6^ Further, *GBA1* screening is more readily available in at‐risk populations, notably the Ashkenazi Jewish (AJ) population, with prenatal screening available in Israel and the United States.^7^ The utilization of future targeted therapies in *GBA1*‐PD as the standard of care may require the integration of genetic testing into routine clinical practice. Variability also exists in the dissemination of information regarding the *GBA1*‐PD link from healthcare providers to patients and their relatives. Further, some commercial entities offer testing for the common coding N370S *GBA1* variant without comprehensive genetic counseling and, importantly, the opportunity to discuss results with a healthcare professional.^8^ Such practices increase the pressure on already overwhelmed publicly or privately funded healthcare services, in this case often leaving practitioners not familiar with the genetics of PD to explain the meaning of a positive *GBA1* variant result.^9^

For patients already diagnosed with PD, *GBA1* testing may provide further information surrounding disease course and treatment options, enable early enrolment into clinical trials investigating gene‐modifying therapies, and inform on family members' risk of harboring *GBA1* variants. For those without a diagnosis of PD, *GBA1* testing can help to answer questions around their potential future risk of PD and enable inclusion into longitudinal studies of prodromal PD markers. The desire for greater knowledge of one's own genes must be balanced with informed consent of the personal and familial implications of a positive *GBA1* variant finding.^10^ Ethical considerations include the utility of identifying *GBA1*‐positive prodromal PD patients in the absence of a proven therapy to slow disease progression. It is important to note the additional ethical considerations in countries where prenatal testing and newborn screening are available and their impact on parents.^11^ Furthermore, *GBA1* variants have variable penetrance, with only a minority of carriers developing PD by 80 years of age.^12^ However, the concept of precision medicine, guided by subject genotype, has gained momentum, and the success of targeted genetic‐based therapies in diseases such as spinal muscular atrophy support this strategy.^13^

Thus, the need for an evidence‐based and clinician‐supported consensus of recommendations on the disclosure of secondary disease associations with *GBA1* variants, namely, PD, is timely, given the initiation of clinical trials for *GBA1*‐PD.^14^ In this article, we review the current knowledge of the link between *GBA1* and PD risk; review the clinical features associated with *GBA1* variants; discuss the practicalities of *GBA1* testing, which is impacted by its pseudogene; and lastly advise how to communicate the risk to index cases and their families.

## *GBA1* ‐PD Association

Substantial evidence demonstrates an excessive burden of LSD gene variants in neurodegeneration.^2^ *GBA1* variants are present in up to 5% of PD cases, with this figure increasing to 15% to 20% in PD patients of AJ origin.^2^, ^15^, ^16^ Notably, other studies have reported a broader range of frequencies of *GBA1* risk variants in PD cases.^17^, ^18^ *GBA1*, encoding the lysosomal enzyme glucocerebrosidase, is involved in sphingolipid metabolism.^2^ *GBA1* is located on chromosome 1q21, encompassing 11 exons and 10 introns. The gene has a 39‐amino acid leader sequence, which has introduced some confusion, because some newer studies now add this on when numbering mutations; that is, N370S is also referred to as p.N409S. Approximately 500 *GBA1* mutations, and numerous nonpathogenic variants for GD (eg, E326K), have been described.^19^ An association between these two phenotypically distinct diseases, GD and PD, has been noted for some time, based on case reports of GD patients and their relatives.^20^, ^21^, ^22^ Subsequently, larger cohort studies and genome‐wide association studies undertaken on PD patients have confirmed *GBA1* as the most important genetic risk factor for PD (overall odds ratio [OR] 5.43).^2^, ^23^

### Variability of PD Phenoconversion Rates

*GBA1* variants exhibit incomplete penetrance for PD, complicating genetic counseling. *GBA1*‐PD penetrance is age specific with PD risk estimates of 1.5% to 4.7% at age 60 years and 7.7% to 9.1% at age 80 years for those harboring *GBA1* mutations.^24^, ^25^ Notably, earlier studies may have overestimated age‐specific PD risk using smaller study numbers showing a 5% to 14% and 15% to 30% risk of PD at 60 and 80 years, respectively.^12^, ^26^, ^27^ Further, each specific *GBA1* variant has a different level of PD pathogenicity with severity determined by its phenotype in homozygous GD patients.^2^ Severe *GBA1* variants (L444P, 84GG, IVS2 + 1, etc.) and mild variants (eg, N370S) increase the risk of PD phenoconversion by ~9‐ to 10‐ and 4‐fold, respectively.^28^ Further, different frequencies of specific *GBA1* variants are found in different populations; N370S is the most common AJ mutation; R496H and 84GG are also mostly found in the AJ population; L444P and R120W in East Asians; and E326K, N370S, H255Q, and D409H in European/West Asians.^28^ A unique genetic signature underlying PD populations has been noted, with a predominance of *GBA1* N396T variants found in the Portuguese, W378G in the French Canadian, K198E in the Colombian, K(−27)R in the Nigerian, and F216L and K(−27)R in the South African PD population.^16^, ^29^, ^30^, ^31^, ^32^ Table 1 outlines the prevalence rates and OR of specific *GBA1* variants in different populations as summarized by Parlar et al,^33^ Zhang et al,^28^ Gabbert et al,^17^ and others. Performing complete sequencing of the entire *GBA1* gene in understudied PD populations, particularly in Latin America and Africa, where the burden of PD has exponentially increased in line with an aging population, may help to define more accurately the mutant *GBA1* alleles and their frequency in the developing world.^56^, ^57^, ^58^ Such work may also highlight factors that contribute to the variable PD phenoconversion rates observed among *GBA1* variant carriers, the majority of whom do not develop PD, and thus inform genetic counseling.^2^

**TABLE 1:** Key *GBA1* variant frequency and associated PD risk

Variants	GD Severity^a^	PD OR	Prevalence of PD in Various Populations, %					References
			African	AJ	American (Latin)	Asian	European/North American	
Frequently investigated *GBA1* variants								
L444P p.(Leu483Pro)	Severe	6.4–30.4	0.4	0.3	2.1	2.6	1.5^b^	17, 28, 33
N370S p.(Asn409Ser)	Mild	2.2–7.8	0.5	13.7	1.1	0.2	1.6	
E326K p.(Glu365Lys)	Benign	1.6–5.5^b^	1.3	1.6	1.1	0.1	4.1	
T369M p.(Thr408Met)	Benign	1.4–5.0	0.9	0.7	0.5	0.0	2.0	
R120W p.(Arg159Trp)	Severe	8.6	0.0	0.0	0.3	0.7	0.1	
Less frequently investigated *GBA1* variants								
84GG c.84dupG	Severe	9.3–13.6	–	2.0–4.0	–	–	0.2–0.3	28, 33, 34, 35, 36, 37
IVS2+1G>A	Severe	8.0–19.1	–	0.7–1.2	0.9	0.9	0.1–1.3	28, 33, 35, 37, 38, 39, 40, 41, 42
K(−27)R p.(Lys13Arg)	Benign	–	6.1–20.0	–	–	–	0.1–0.4	16, 29, 30, 36, 43, 44, 45
H255Q p.(His294Gln)	Severe	4.8	–	–	–	–	0.7–3.4	28, 38, 46
D409H p.(Asp448His)	Severe	3.8	–	–	–	0.2–1.1	0.1–3.4	28, 35, 45, 46, 47, 48, 49, 50, 51, 52
R496H p.(Arg535His)	Mild	4.4–7.3	–	0.6–2.1	–	0.2	0.1	28, 33, 35, 39, 47, 52, 53
V394L p.(Val433Leu)	Severe	4.9–6.7	–	0.2–1.0	–	–		33, 37, 54
Rec*Nci1* p.[(Leu483Pro;Ala495Pro; Val499Val)]	Severe	3.2–7.3	–	0.5	–	0.2–2.6	0.1–1.0	28, 33, 36, 47, 53, 54, 55

*Note*: Data are summarized by Gabbert et al,^17^ Parlar et al,^33^ and Zhang et al.^28^ Other studies were used for data on less frequently investigated *GBA1* variants.

Abbreviations: PD, Parkinson's disease; GD, Gaucher's disease; OR, odds ratio; AJ, Ashkenazi Jewish.

^a^ Effect of *GBA1* variant on the severity of GD. *GBA1* variants that cause nonneuronopathic type 1 GD are termed *mild*. Severe *GBA1* variants cause type II and III neuronopathic GD, and benign variants refer to those that are not pathogenic for GD but do increase the risk of PD.

^b^ Notably, a lower OR for the E326K *GBA1* variant of 1.97 was reported by Zhang et al.^28^ Also, a higher prevalence of L444P *GBA1* variant has been noted in European populations by other studies.^18^

### Clinical Features of Prodromal and Established *GBA1* ‐PD

The search for an accurate and reliable method of early detection, preferably prediction, of PD phenoconversion in asymptomatic *GBA1* variant carriers is of critical importance. Significant neurodegeneration precedes the onset of motor symptoms in PD, typically emerging after ~60% to 70% dopaminergic neuronal loss in the substantia nigra.^59^ Ultimately, the goal of identifying individuals with either a genetic predisposition and/or clinical features considered part of the PD prodrome at the earliest opportunity is to offer targeted neuroprotective therapies that can either (1) delay disease progression or, ideally, (2) enable primary prevention of clinical PD.

Follow‐up of asymptomatic carriers of *GBA1* variants represents an opportunity to recognize early prodromal PD features in those who undergo phenoconversion. Longitudinal clinical, neuroimaging, and biochemical characterization of this patient subgroup may highlight candidate biomarkers demonstrating predictive, diagnostic, and prognostic accuracy in PD.^60^ Robust biomarkers may enable the stratification of PD patients into more homogeneous subgroups in trials, a factor that may have hindered the identification of a reliable biomarker so far.^60^ Interestingly, the PD prodrome may precede diagnosis by more than 20 years.^61^ Screening of prodromal PD patients has demonstrated an initial olfactory loss followed by other nonmotor symptoms such as constipation and erectile dysfunction (starting 10–16 years before diagnosis), in addition to urinary dysfunction and cognitive decline (7–9 years before phenoconversion).^61^ A prominent feature of prodromal neurodegenerative synucleinopathy is rapid eye movement sleep behavior disorder (RBD), with more than 80% of individuals with RBD eventually developing PD or dementia with Lewy bodies.^62^ Depression, RBD, and hyposmia occur at an increased incidence in *GBA1* variant carriers without PD compared with noncarriers.^63^, ^64^ Multimodal characterization of genetic cohorts at risk of PD is of importance for the timely implementation of future neuroprotective therapies, serving also to inform significant disease processes in idiopathic PD. Indeed, several longitudinal investigations of *GBA1* variant carriers are under way, not limited to the UK‐based RAPSODI and PD FRONTLINE study, Parkinson's Progression Markers Initiative, and active clinical trials aiming to evaluate prodromal parkinsonian features in this unique cohort (ClinicalTrials.gov Identifier: NCT05253560). Shared biorepositories and data from ethnically diverse prodromal PD patients are necessary to aid such work.^60^

*GBA1*‐PD patients show some alterations in disease course and clinical phenotype compared with those with idiopathic PD. An earlier onset by ~2 to 10 years accelerated progression of motor and cognitive decline, and poorer survival rates occur in *GBA1*‐PD cohorts compared with their sporadic counterparts.^34^, ^65^, ^66^ *GBA1* variants harbor a dose and variant‐type effect, with an earlier PD onset also reported in patients with biallelic (homozygous or compound heterozygous) *GBA1* variants relative to heterozygotes,^67^ and in carriers of severe variants (5 years earlier) compared with mild variants.^38^, ^68^ Stratifying by *GBA1* variant severity, cases with severe *GBA1* variant‐associated PD more often exhibit a higher burden of motor symptoms; earlier, more frequent nonmotor symptoms; and overall a more aggressive disease course compared with mild variants.^3^, ^38^ However, the correlation is not always as straightforward, with similar clinical profiles sometimes seen in those who carry less severe variants.^3^ Risk/benefit analyses have been discussed on the clinical utility of subthalamic nucleus deep brain stimulation in *GBA1*‐PD cohorts given mixed reports suggesting a potential worsening in cognitive decline.^69^, ^70^, ^71^ Comprehensive genetic counseling for *GBA1* variants ideally should address prognostic in addition to predictive information in communications to patients and their relatives, stressing the known incomplete penetrance.

## Should *GBA1* ‐PD Counseling Be Included in Standard Clinical Care?

Systematic screening for *GBA1* variants may inform PD prognosis in affected individuals, quantify recurrence risk to relatives, enable longitudinal characterization of prodromal PD, and perhaps predict future PD phenoconversion. In addition, it would assist in the implementation of accurate patient stratification as a prerequisite in PD trials. The latter may facilitate investigations into the utility of *GBA1* as a therapeutic target.^3^ Testing for *GBA1* variants has largely remained confined to specialist neurology centers, typically not being offered or accessible to most PD patients, especially late‐onset PD.^72^ Newer large‐scale initiatives, such as the ROPAD and PD GENEration study, aim to make genetic testing and counseling more available to PD patients and have already enrolled more than 30,000 patients globally.^5^, ^73^ With several *GBA1*‐targeted therapies in the late stages of clinical trials, the shift toward more routine use of genetic testing among the PD population will become of paramount importance.^5^, ^73^, ^74^ However, factors impeding the delivery of appropriate and relevant genetic counseling for *GBA1*‐PD pertain largely to the variable penetrance of PD associated with *GBA1* variants, in tandem with differences in *GBA1* sequencing and a lack of guidance for the interpretation of PD risk associated with specific *GBA1* variants.^24^, ^26^ Existing literature has provided some guidance,^75^, ^76^ but greater clarity is required to enhance the clinical utility of *GBA1* testing and clarify counseling guidelines defining and explaining (a) PD risk with specific *GBA1* variants; (b) family counseling specifically in prenatal tests; and lastly, but not least, (c) GD counseling in incidental findings of homozygotes/compound heterozygotes.

### Practicalities of *GBA1* Testing

Shortcomings in *GBA1* sequencing have hindered widespread delivery of genetic counseling. Challenges persist in identifying *GBA1* variants because of the nearby pseudogene *GBAP1*.^77^ A high homology of 96% is present between *GBA1* and *GBAP1*, increasing to 98% between intron 8 and 3′ untranslated region.^77^ This predisposes to reciprocal and nonreciprocal recombination, generating complex alleles.^78^ Recombinants in repetitive or highly homologous regions cannot be easily recognized by short‐read next‐generation sequencing methods that fail to map to the reference genome.^79^ Long‐read Oxford Nanopore Technologies MinION sequencing, the new Gauchian bioinformatics tool, computational customized scaffolds assisting read alignment, and improved choice of polymerase may overcome such obstacles.^17^, ^80^, ^81^, ^82^ A recent study demonstrated that the algorithm, Gauchian, was able to detect structural variants and single‐nucleotide variants in *GBA1* with Oxford Nanopore Technologies methods capable of detecting recombinants.^80^ Notably, false negatives and false positives may still be generated with new technologies, impacting genetic counseling.^80^ In summary, genomic sequencing technology is rapidly advancing; the refinement of *GBA1* sequencing to increase the capture of recombinant alleles, thereby enabling comprehensive genomic analyses, will inform genetic counseling.^80^

Large‐scale, full sequencing of the entire *GBA1* gene is essential to identify variants accurately, especially to show the clinical relevance of novel, rare, and population‐specific *GBA1* variants.^83^ However, most studies have screened for selected, common GD pathogenic *GBA1* variants, leaving a proportion of carriers undiagnosed and incorrectly deemed noncarriers.^83^ Highlighting this issue, a meta‐analysis indicated that only 4 of 11 *GBA1*‐PD cohorts in Latin America (n = 735, controls = 445) underwent full *GBA1* sequencing.^56^ Notably, estimates for *GBA1*‐PD prevalence and OR in Brazil, the most populated country in Latin America, have been generated entirely using incomplete sequencing.^56^ The list of known GD‐causing and non–GD‐causing *GBA1* variants in PD has grown considerably since recognition of the *GBA1*‐PD association. A greater understanding of the mechanisms behind GD versus PD‐related *GBA1* sequence changes is needed. Databases such as those created by Parlar et al^33^ endeavor to assist the sharing of genomic data and make information more accessible (https://pdgenetics.shinyapps.io/GBA1Browser/); complete analyses of the *GBA1* gene are likely to elucidate further phenotype–genotype associations, adding to the evidence base.

### Counseling for the *GBA1* ‐PD Association

#### Status Quo, Knowledge, and Interest in Genetic Testing

Counseling of the PD predisposition risk in nonmanifesting *GBA1* carriers or the prognostic trajectory in PD patients with a recently identified *GBA1* variant has been limited for many years by significant uncertainty over the pathogenicity associated with specific variants. Recent evidence has been informative on the lifetime risk of PD and PD severity associated with specific *GBA1* variants, albeit the latter still requires further clarification.^28^ There remains no established standardized practice of communicating secondary disease associations, such as PD, to carriers of *GBA1* variants.^84^ There is also no consensus addressing when and how genetic counselors and clinicians should initiate discussions of the risk of developing PD with GD patients given its relatively low lifetime risk and larger primary disease concerns relating to GD.^84^

With no preventative or disease‐modifying treatment for PD currently available, the rollout of routine genetic testing in PD clinics will, in part, depend on demand from patient groups. Among PD patients, a lack of awareness of the connection between GD and PD has been noted.^84^ However, PD probands and relatives have continuously expressed an interest in genetic testing.^11^, ^85^ Most GD patients wish to be informed of the increased PD risk by their physician at the time of GD diagnosis alongside other potential secondary disease associations.^86^ Pretest counseling of the *GBA1*‐PD link is also unlikely to reduce screening uptake, with study participants indicating such knowledge would be beneficial and would not add any further anxiety after being given the results of a positive *GBA1* variant finding.^11^

#### Interpreting and Communicating PD Risk

Limited knowledge, a lack of professional guidelines, and inexperience in initiating discussions surrounding PD risk have all been cited as reasons behind inconsistent discussions of this link with carriers of *GBA1* variants.^10^, ^87^ Patient autonomy, enabled by improved education of this link, is a central tenet of high‐quality patient care. Standard clinical practice should entail the routine dissemination of information regarding the lifetime risk of PD to both heterozygous and biallelic *GBA1* carriers directly from clinicians and trained genomic counselors (1) before *GBA1* testing to ensure informed consent, and (2) at diagnosis after the establishment of patient preference for genetic result disclosure.

When counseling for a *GBA1* variant, a brief explanation of genetics is recommended. PD risk is defined by the association between specific *GBA1* variants and GD phenotype: low risk, *GBA1* variants that are associated with PD, but not GD (E326K, T369M); moderate/high risk, *GBA1* variants that are associated with GD and PD (N370S); and unknown significance, either novel variants or those with insufficient evidence to determine pathogenicity.^88^ Contextualizing ORs assigned to each *GBA1* variant to patients can be complex.^33^ Accounting for the lifetime risk of PD in the general population (~3%) and a recent meta‐analysis of *GBA1* variant ORs by Zhang et al,^28^ PD pathogenicity of specific *GBA1* variants can be explained by the number of people out of 100 with such a variant to develop PD.^28^, ^89^ For instance, to counsel for the PD pathogenicity of the *GBA1* L444P variant, it is possible to state that “approximately 26 individuals out of 100 with this genetic change are likely to develop PD by age 80. Whilst this is a higher figure than the general population (3 out of 100), most people with this GBA1 variant will never develop the disease.”^90^ It is important to provide statistics on PD risk in context, stressing that a sizable majority of carriers of *GBA1* variants never undergo PD phenoconversion. Although there is still no reliable method to predict in whom PD phenoconversion will occur, several investigations are under way.^91^ A transcript of *GBA1* variant counseling for heterozygous *GBA1* variant carriers is included in Table 2.

**TABLE 2:** Transcript for *GBA1*‐PD counseling in heterozygous *GBA1* variant carriers

**Ideas, concerns, expectations**
Confirm patient details with three identifiers (name, date of birth, address). Provide the patient with time to ask questions. Re‐explain the purpose of the study. Ask if patient has any concerns or expectations from the study or their test results, including their understanding of and benefit from either a positive or a negative result, and how they might share this with their family. Ask for consent to discuss genetic test results.
2 **General information regarding genetics**
We all have two copies of each gene, one from each parent (and we have ~25,000 genes). Genes are responsible for giving us specific characteristics such as hair and eye color and disease susceptibility. Even a single change (known as a variant) in our genes can affect our risk of disease. Regarding PD: Approximately 3 in 100 people in the general population will develop PD by the age of 80 years. *GBA1* is responsible for GD when two copies of the gene are affected. Changes in *GBA1* can also be associated with a small increased risk for PD (even if just one copy is changed). Only a small minority of people with *GBA1* changes will develop PD (5%–10% of individuals).
3 **Results disclosure if *GBA1* variant identified**
In your case, we have found that one of the two copies of the *GBA1* gene carries a genetic change. This specific genetic change *slightly increases* your PD risk compared with those who have two normal *GBA1* copies. The evidence we have of this stems from past studies (we are in the process of gathering longitudinal data). The PD risk is relatively small. To give you an idea, in the general population, 3 of 100 people develop PD by 80 years of age. With the specific variant you carry your risk is as follows: **Table:** Variants Number (as a range) out of 100 people who will likely develop PD by the age of 80 y (based on ORs)^a^ Number (as a range) out of 100 people who will likely develop PD by the age of 80 y (based on the 95% CI of ORs)^a^ N370S 11 9–14 E326K 6 5–7^b^ T369M 5 4–8 L444P 26 19–36 Most individuals with this specific *GBA1* variant will not develop PD. It is impossible to predict who will develop PD. You will not develop GD because you would need a change in both copies of your *GBA1* gene. For those with PD, the presence or not of a *GBA1* variant does not influence their treatment. Current trials are recruiting patients with *GBA1* variants. Faster progression of PD has been noted. There are mixed reports of worsening cognitive decline after subthalamic nucleus deep brain stimulation.
4 **Results disclosure if *GBA1* variant of unknown significance identified**
Not many carriers of this specific variant have been reported in the literature. The estimate of PD risk and clinical effect is unknown. Case reports of carriers with this variant may in time demonstrate its associated PD risk. It may be possible to return to these individuals in the future with further information once more data have emerged.
5 **Risk to family members**
Ensure there is a discussion regarding the benefit versus risk of disclosing this genetic result to relatives, providing the patient with sufficient information relating to their genetic risk to make an informed decision. Provide support if patient wishes/does not wish to share genetic result with relatives. Offer to assist in counseling family members regarding their genetic risk on behalf of or in conjunction with the patient. Children For children to develop GD, they need to receive a copy of the abnormal gene from both parents. In the general population, the probability of both parents having changes in the same gene is extremely rare. If you are a carrier of a *GBA1* change and you are planning to have children, you *should contact your general practitioner*, and your partner might be tested and/or referred to a genetic counselor: If both parents carry a change in the *GBA1* gene: 25% chance of having a child with GD 50% chance of having a child that is a carrier of a *GBA1* variant (but does *not* have GD) If you have a *GBA1* variant but your partner does not: 0% chance of having a child with GD 50% chance of having a child that is a carrier of a *GBA1* variant If found to be a *GBA1* variant carrier, there are associated risks of developing PD (risk is listed earlier)
6 **Closing**
Summarize. Take questions. Provide information in writing. Offer follow‐up consultation.

Abbreviations: PD, Parkinson's disease; GD, Gaucher's disease; OR, odds ratio; CI, confidence interval.

^a^ Number (out of a range) out of 100 people who will develop PD by age 80 y with each specific *GBA1* variant calculated by multiplying the 95% CI of the best estimated OR or the best estimated OR itself of the variant (from Zhang et al,^28^) with the average risk of PD in the general population (3/100).

^b^ Higher ORs were found in people of Ashkenazi descent.

Following the diagnosis of a *GBA1* variant, individuals may have additional concerns regarding the PD risk of adult relatives. *GBA1* variant carriers should be invited to decide whether to disclose genetic results to family members, enabling them to be informed on the presence of increased PD risk in the family, and decide whether to undergo *GBA1* testing themselves. Indeed, desire for genetic testing increased with hypothetical risk of PD among relatives.^92^ Relatives listed early identification of a *GBA1* variant to be eligible for current and future PD prevention and neuroprotection trials as important in their decision to be tested.^92^ Earlier diagnoses of asymptomatic *GBA1* variant carriers would enable inclusion into longitudinal investigations of prodromal PD markers, aiding characterization of the prodrome and the search for validated biomarkers of progression to PD.

#### Ethical Considerations

The expansion of genetic testing for incurable neurodegenerative disorders for diagnostic and predictive purposes has highlighted key ethical considerations. Extensive, pretest counseling should occur, ideally in person over several clinic visits, explaining the associated benefits, risks, and limitations of *GBA1* testing.

Effective pretest counseling should delve into the various implications of a positive *GBA1* variant finding. Prior studies into patient opinion on genetic counseling showed that 87% of individuals wished to be informed of the increased PD risk before GD carrier screening, noting that in 93% of individuals this information would not have made them less likely to engage with screening.^11^ Further, *GBA1* pretest counseling should be tailored to the individual undergoing testing. For instance, pretest counseling may center on individual PD risk in asymptomatic *GBA1* variant carriers and the option of enrolment into studies on prodromal PD markers, whereas for individuals already diagnosed with PD, genetic counselors may find discussions relating to prognosis, treatment, and clinical trials on gene‐modifying therapies more appropriate. Further, *GBA1* counseling should be inclusive of discussions on disclosing genetic results with relatives and its impact on (1) their own PD risk, and (2) the risk of GD and PD in offspring of those of childbearing age and the potential future risk of GD and PD for the offspring of the children of carriers. The latter would require further referral for genetic counseling and testing in potential parents.

Estimating risk for neurodegenerative disease is relatively straightforward with high‐penetrance genes (eg, Huntington's disease).^93^ Disease risk is more difficult to quantify in PD, where disease penetrance is variable. Communication of challenges in estimating genetic risk and incomplete penetrance to individuals undergoing *GBA1* testing are essential. Genetic counselors should address potential psychological harm and genetic discrimination in social, healthcare insurance, and employment settings following the finding of an abnormal genetic result.^94^ Exploring such issues with people will help to inform patients on the benefits and risks of genetic testing, especially for diseases for which we have yet to yield successful treatments.^95^

In addition, advances in genomic science have led to the commercialization of genetic testing.^94^ Direct‐to‐consumer (DTC) genetic screening tests for risk of breast/ovarian cancer, Alzheimer's disease, PD, and many other diseases are now readily available, including testing of the *GBA1* gene.^94^ Ethical concerns have been raised given DTC genetic testing companies are not obliged to provide counseling after the disclosure of genetic results, potentially leading to negative outcomes, such as engaging in unnecessary or adverse health decisions, and psychological distress, especially in the case of severe and incurable neurodegenerative diseases. Neurogenetic testing is likely to become more commonplace; tighter regulation to guide the practice of DTC genetic screening services is urgently required.

#### Other Important Considerations

Potential genetic modifiers of *GBA1* may play a role in modulating susceptibility toward developing PD.^96^ The role of *PSAP*, *SCARB2*, *LRRK2*, *SMPD1*, *CTSB*, CTSD, *GRN*, and others in modifying PD risk continues to be fully evaluated.^91^, ^96^, ^97^ Potential sex‐specific differences in *GBA1*‐PD also need to be fully understood and included in comprehensive genetic counseling.^98^, ^99^ Additional factors potentially influencing penetration are under active investigation, including the microbiome and other environmental modifiers.^100^

## A Global Outlook on Genetic PD

Ensuring comprehensive genetic sequencing of understudied PD populations, particularly in Asia, Latin America, and Africa, would greatly aid PD research and provide genetic counseling, which is adjusted based on ethnic risk.^28^ Such work would ultimately facilitate the application of precision medicine. In this review, we provide a brief outlook of investigations of genetic PD in Africa to highlight this unexplored area of PD research.

### A Focus on Africa and Equity of Access to Genetics Healthcare

Reports from the World Health Organization estimate an overall life expectancy in Africa of 61 years in 2018, compared with 46 years in 2000.^101^ Improved access to healthcare, intensive public health campaigns, and increased availability of treatments for transmissible diseases (notably malaria, tuberculosis, and HIV) have accounted for this increase in life expectancy.^101^ As a result, the burden of noncommunicable disorders, such as diabetes, ischemic heart disease, stroke, and indeed PD, is increasing in this aging African population. The clinical features and genetics of PD in Africa, particularly Sub‐Saharan Africa, have been poorly described.^57^ Difficulties hindering African PD research are substantial and include a lack of insurance coverage of chronic conditions and critically levodopa medication, underdeveloped infrastructure (with few laboratories capable of genomic analyses), disease stigma discouraging the disclosure of a family history of PD, and a limited number of neurologists.^57^ Neurological services are scarce and unavailable in some regions.^102^ A survey published in 2022 found that 34 of 54 countries on the African continent have less than 10 neurologists, including Angola, Ghana, Uganda, and Zimbabwe.^102^ African PD research has remained largely uninvestigated; however, initial reports have suggested the K(−27)R *GBA1* variant is more frequently detected in this population, albeit its link to PD risk is yet to be confirmed.^29^, ^30^ Moreover, a genome‐wide association study of ~200,000 individuals of African and African admixed populations, conducted by the Global Genetics Program (GP2), recently identified a novel PD risk factor in intron 8 of the *GBA1* gene (OR 1.58).^103^ Further initiatives such as this and the inclusion of diverse groups in future studies would be greatly beneficial for PD research globally.

## Conclusion

Availability and uptake of neurogenetic screening tests have increased exponentially in recent years, both in clinics and DTC testing companies. Although representing an exciting opportunity, education on genetic risk, testing benefits, and limitations is necessary to ensure individuals and physicians are prepared to discuss interpretation of incidental genetic findings, such as the identification of a *GBA1* variant. Furthermore, the availability, cost, cost–benefit ratio, access to counseling of patients and relatives, and education of physicians need to be considered alongside the ethics and practical implications of *GBA1* genetic testing.

There is now the potential for the inclusion of *GBA1* testing and, thus, *GBA1* variant counseling in standard clinical care. However, there remains a pressing need for guidelines to aid the standardization of genetic counseling for *GBA1* variants, which currently vary according to center and clinician. Collaboration across regional neurology services will be required to create a set of guidelines to inform *GBA1* counseling.

## Author Roles

(1) Research project: A. Conception, B. Organization, C. Execution; (2) Manuscript Preparation: A. Writing of the First Draft, B. Review and Critique.

S.R.L.V.: 1A, 1B, 1C, 2A, 2B.

A.H.V.S.: 1A, 1B, 1C, 2B.

R.M., M.T., S.L.D.P., E.M., S.M., S.Y., Naomi Limbachiya, S.K., Nadine Loefflad, G.J.L., Z.G.‐O., R.N.A., E.S.: 2B.

## Financial Disclosures

E.S. and G.J.L. are supported by the Intramural Research Programs of the National Human Genome Research Institute and National Institutes of Health. Z.G.‐O. received consultancy fees from Lysosomal Therapeutics Inc. (LTI), Idorsia, Prevail Therapeutics, Ono Therapeutics, Denali, Handl Therapeutics, Neuron23, Bial Biotech, Bial, UCB, Capsida, Vanqua Bio, Congruence Therapeutics, Takeda, Jazz Pharmaceuticals, Guidepoint, Lighthouse, and Deerfield. R.N.A. has received grants from Parkinson's Foundation for acting as a Steering Committee Member; receives funding from the National Institutes of Health, Department of Defense, The Michael J. Fox Foundation, and the Silverstein Foundation for GBA/PD; and has received consulting fees from Biogen, Biohaven, Capsida, Gain Therapeutics, Sanofi, Servier, Takeda, and Vanqua Bio. A.H.V.S. is employed by University College London and has received grants from MRC (UK), Cure Parkinson's, The Michael J, Fox Foundation, and Aligning Science Across Parkinson's. A.H.V.S has received royalties from OUP, Wiley, and Cambridge University Press, and has received consultancy fees from Capsida, Neuron23, Bial, and Congruence. S.L.D.P. declares honoraria from Moderna. R.M., E.M., Naomi Limbachiya, S.K., S.Y., Nadine Loefflad, M.T., S.M., S.R.L.V. declare no conflicts of interest nor financial disclosures.

## Data Availability

Data sharing is not applicable to this article as no new data were created or analyzed in this study.
